# First salvage treatment with bendamustine and brentuximab vedotin in Hodgkin lymphoma: a phase 2 study of the Fondazione Italiana Linfomi

**DOI:** 10.1038/s41408-019-0265-x

**Published:** 2019-12-11

**Authors:** A. Broccoli, L. Argnani, B. Botto, P. Corradini, A. Pinto, A. Re, U. Vitolo, S. Fanti, V. Stefoni, P. L. Zinzani, on behalf of Fondazione Italiana Linfomi ONLUS

**Affiliations:** 1 Institute of Hematology “L. e A. Seràgnoli”, University of Bologna, Bologna, Italy; 2 Hematology, Department of Oncology, University Hospital Città della Salute e della Scienza, Torino, Italy; 3 IRCCS Istituto Nazionale dei Tumori, University of Milano, Milano, Italy; 4 Hematology-Oncology and Stem Cell Transplantation Unit, National Cancer Institute, Fondazione Pascale, Napoli, Italy; 5 Hematology, Azienda socio-sanitaria territoriale, Spedali Civili di Brescia, Brescia, Italy; 6 Nuclear Medicine, Sant’Orsola-Malpighi University Hospital, University of Bologna, Bologna, Italy

**Keywords:** Haematological diseases, Clinical trial design

## Abstract

Effective salvage options inducing high complete metabolic response (CMR) rates without significant toxicity are needed for Hodgkin lymphoma (HL) patients failing induction treatment and who are candidate to autologous stem cell transplantation (ASCT). Brentuximab vedotin (BV) and bendamustine are active monotherapies in the relapsed/refractory setting and their combination (the BBV regimen) possibly enhances their activity. This single-arm multicenter phase 2 study investigated the efficacy and safety of BBV as first salvage therapy in 40 patients with relapsed/refractory HL. Thirty-eight patients were evaluable for efficacy: 30 (78.9%) had a CMR and 2 (5.3%) a partial response, leading to an overall response rate (ORR) of 84.2%. The ORR in the primary refractory subset was 75.0%, among relapsed patients it was 94.4%. Thirty-five patients could mobilize peripheral blood stem cells and 33 underwent ASCT. At a median follow-up of 23 months, the estimated 3-year overall survival and progression-free survival are 88.1% and 67.3%. During therapy, only 3 grade IV cases of neutropenia occurred and resolved within a week. No grade 4 extrahematologic toxicities were reported; skin reactions were however rather frequent (65%). These results suggest that the BBV regimen exhibits promising efficacy and a manageable toxicity in a challenging subpopulation of HL patients.

## Introduction

The standard treatment for patients with classical Hodgkin lymphoma (cHL) who are unresponsive to upfront therapy or relapse after primary treatment consists of salvage chemotherapy (aimed at harvesting autologous stem cells from peripheral blood), followed by high-dose chemotherapy and autologous stem cell transplantation (ASCT). This latter phase has to be reserved only to those patients who are able to tolerate a highly toxic conditioning and a fairly prolonged myelosuppression.

Although this approach has yielded a long-term progression-free survival (PFS) in 50–60% of patients with chemosensitive relapse^1,2^, outcomes remain poor in those with primary chemorefractory disease, where long-term survival rarely exceeds 15–17%^3,4^. Disease recurrence still remains the principal cause of ASCT failure, and early disease progression after transplant, i.e., within 6 months from high-dose conditioning, emerges as a clear predictor of unfavorable outcome^4^.

Under this light, optimization of the outcomes obtained with high-dose regimens and ASCT still remains a priority, which is required to offer the best chance of cure for the largest fraction of patients with refractory and relapsing disease^5^. In particular, any strategy aimed at achieving a minimal disease status, and at specifically obtaining a positron emission tomography (PET)-negative status before ASCT without severe toxicity, would represent a major advance in the overall management of these patients^6–9^.

Brentuximab vedotin (BV) potentially induces deep responses when applied in the context of a first salvage treatment before ASCT, even if as single agent^10^. Importantly, BV displays a favorable toxicity profile, without significant myelosuppression and with no cross-resistance with most of the agents employed during induction or high-dose conditionings. Moreover, BV induces PET negativity in patients with advanced disease, with clinical responses observed rather rapidly, i.e., within the first 3–4 cycles in most responding subjects, allowing the timely application of the transplantation procedure^11,12^. Besides BV, the “freshly rediscovered” old bendamustine has shown clinical activity in patients with multitreated cHL^13^, and may also overcome the resistance to previous BV treatment^14^. Both BV and bendamustine can be administered on an outpatient basis, they are well tolerated in terms of hematological adverse events (AEs) and do not show overlapping toxicities^11,13^. For this reason, there is a strong rationale to combine favorably these two agents in order to exploit a synergistic effect with the purpose of improving the remission rates observed with either agent in the pre-ASCT setting. LaCasce and coworkers presented some preliminary data on BV and bendamustine combination in 2014 at the American Society of Hematology Meeting: the phase 1 part of their study was designed to determine the recommended dose of bendamustine associated with BV. No dose-limiting toxicities were observed during the trial, thus the authors concluded that a standard dose of 1.8 mg/kg of BV could be safely combined with 90 mg/m^2^ of bendamustine^15^.

Here, we present the results of a phase 2 study evaluating the efficacy and safety of the bendamustine plus BV (BBV) regimen applied as first salvage strategy in patients with cHL. The study protocol also contemplated the enrolment of patients with relapsed or refractory CD30^+^ peripheral T-cell lymphomas in a separate cohort, for which enrolment is still ongoing.

### Patients and methods

#### Study oversight and patient population

This was a single-arm, open-label, multicenter, phase 2 clinical trial, aimed at evaluating the antitumor efficacy and safety of the BBV combination as first salvage therapy in patients with relapsed or refractory cHL. The study involved seven Italian hematology centers adhering to the Italian Lymphoma Foundation (Fondazione Italiana Linfomi, FIL). The trial was conducted in accordance with the Declaration of Helsinki and the study protocol was approved by the Ethical Committee of the Coordinating Center (Comitato Etico di Bologna) and by the Ethical Committee of each participating site. Written informed consent was obtained by patients before any study procedure. The trial was registered at www.clinicaltrials.gov, NCT02499627, and given the EudraCT number 2014–005382–79.

Patients aged 18–60 years were eligible if affected by histologically confirmed CD30^+^ cHL at first relapse or with primary refractory disease (i.e., having received only one previous line of treatment). Patients should have never received either bendamustine or BV during induction and have never undergone ASCT. Fluorodeoxyglucose avid and measurable disease documented by both PET and computed tomography (CT) was required for enrolment. Repeat biopsy was not considered mandatory before entry into the trial, and rebiopsy was at the discretion of the treating physician. Results of CD30 expression from the most recent postdiagnostic biopsy of relapsed/refractory disease was obtained from pathology reports of a tumor block (biopsy taken at diagnosis or relapse) to enable study enrollment (per inclusion criteria). Central review for CD30 expression (using BerH2 antibody) was performed on diagnostic histological material. Diagnosis of HL was central reviewed by an expert pathologist and his staff.

The applied salvage regimen consisted of intravenous bendamustine, administered at a dose of 90 mg/m^2^ on day 1 and 2, and BV, given at the total dose of 1.8 mg/kg on day 1 of each 21-days-based cycle. Drug premedication was mandatory and included corticosteroids and antihistamines.

**Fig. 1:**
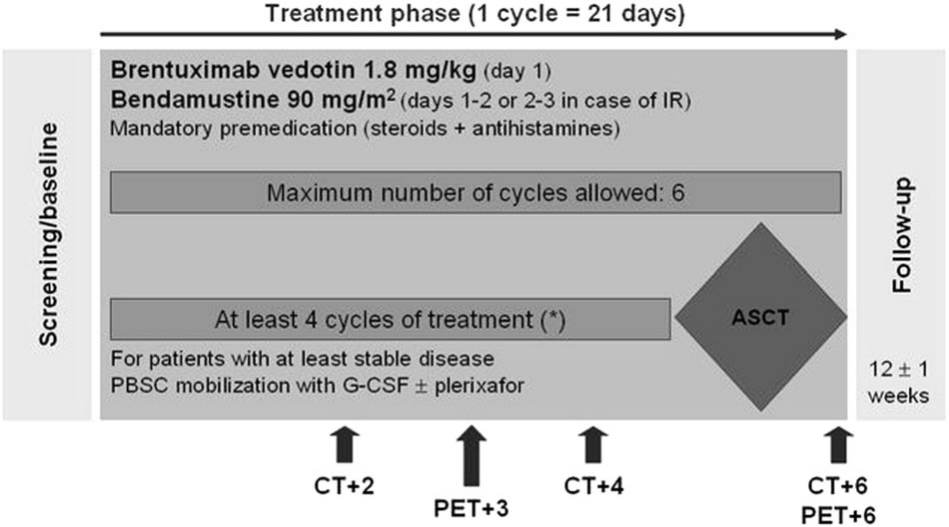
Treatment plan and timing of CT and PET scan assessments. (*) Patients in response were allowed to undergo ASCT after 2 cycles in case of severe toxicity (see text for details). IR, infusion reaction; ASCT, autologous stem cell transplantation; PBSC, peripheral blood stem cells; and G-CSF, granulocyte colony-stimulating factor.

Up to 6 cycles of the combination regimen were allowed (Fig. 1). All patients achieving at least a disease stability could be considered eligible to peripheral blood stem cell (PBSC) mobilization (performed with granulocyte colony-stimulating factor [G-CSF] and plerixafor, if required) and could proceed to ASCT at any time beyond cycle 4. Patients in response were allowed to undergo ASCT after 2 cycles, if they experienced any toxicity that would prevent a safe delivery of subsequent cycles, according to physician’s judgement. Treatment was concluded in case of disease progression or development of unacceptable toxicity, whichever came first.

### Study assessments

The anticancer activity of this regimen was assessed according to the Lugano Classification at the end of the combination treatment^16^. ASCT, if performed, was not intended as part of the study protocol. Dedicated CT scan of neck, chest, abdomen, and pelvis (with and without contrast) was performed at baseline, after cycles 2 and 4, and within 30 ± 7 days after cycle 6 or early study withdrawal. PET scan was also repeated after cycle 3 and within 30 ± 7 days after cycle 6 or early study discontinuation (Fig. 1). For patients proceeding to ASCT after cycle 2 or 4, a pre-ASCT PET scan had to be performed before conditioning, provided this had not been done earlier than 6 ± 1 weeks. PET scans were scored according to the Deauville five-point scale^17^ and were centrally reviewed.

### Study endpoints and statistics

The primary endpoint of the study was the overall response rate (ORR) and the complete metabolic response (CMR) rate to the combination regimen. Secondary endpoints were represented by PFS, overall survival (OS) and disease-free survival (DFS) at 1 year. PFS and OS were calculated for all patients since the entry onto study; DFS was determined in all CMR patients from the moment in which a response was documented to the first determination of relapse, progression, or death from any cause. All patients receiving at least 2 cycles of the treatment and undergoing disease status reassessment by both CT and PET scan constituted the efficacy-evaluable population.

Sample size estimation was performed by Fleming's single-stage procedure. Defined p0 as the proportion of response below which the treatment does not warrant further investigations and pa as the proportion of responses beyond which a phase 3 trial should be carried out, we set p0 = 0.5 and pa = 0.8. The number of patients required, given a type I error (alpha) at 0.05 one sided and a power of 1-beta = 80%, is 36 and the number of successes 28. Taking into account a drop out of 10%, the number of patients was set at 40.

Safety assessment was performed in all patients receiving at least one dose of the combined treatment. The severity of AEs was graded according to the National Cancer Institute Common Terminology Criteria for AEs, version 4.03. Additional analyses were addressed to establish the feasibility of PBSC collection, in terms of percentage of success and amount of PBSC harvested.

Demographics and patients’ characteristics were summarized by descriptive statistics (median and range), and survival functions were estimated by using the Kaplan–Meier method. Statistical analyses were performed with Stata 11 (StataCorp LP, TX).

## Results

Forty patients with refractory (*N* = 20, 50%) or relapsed (*N* = 20, 50%) cHL were enrolled in the study between January 2016 and December 2017, at a median time of 7 months (range: 5–67), since disease diagnosis. Median age at the beginning of the salvage treatment was 38 (range: 20–59) years. Eight patients displayed extranodal involvement, including bone (three patients), lung (three patients), liver (one patient), and liver and lung (one patient). Induction treatment consisted of doxorubicin, bleomycin, vinblastine, and dacarbazine in all cases. In one patient, doxorubicin was administered as a liposomal formulation. The median number of BBV cycles received was 4.

### Response to treatment and stem cell mobilization

Thirty-eight patients were assessable for disease response at the end of the BBV regimen. Two patients, in fact, displayed disease progression before the first CT scan reassessment. An objective response was observed in 32 of the evaluable patients (84.2%), with 30 of them obtaining a CMR (78.9%) and 2 a partial response (PR; 5.3%). A detailed breakdown of Deauville scores along with the associated clinical response is given in Table 1. The ORR in the primary refractory subset was 75.0% (with a CMR rate of 65.0%), while among relapsed patients it reached 94.4% (all patients in CMR). If the whole patient population intended to receive the treatment were considered (all the 40 patients enrolled), the ORR would be 80%, with a CMR rate of 75% and a PR rate of 5%.

**Table 1:** Clinical responses according to the Deauville five-point scale.

	Patients (*N* *=* 38)	%
*Complete metabolic response*		
Deauville 1	5	13.1
Deauville 2	19	50.0
Deauville 3	6	15.8
*Partial response*		
Deauville 4	2	5.3
*No metabolic response/stable disease*		
Deauville 4	1	2.6
Deauville 5	1	2.6
*Progressive disease*		
Deauville 4	1	2.6
Deauville 5	3	7.9

**Fig. 2:**
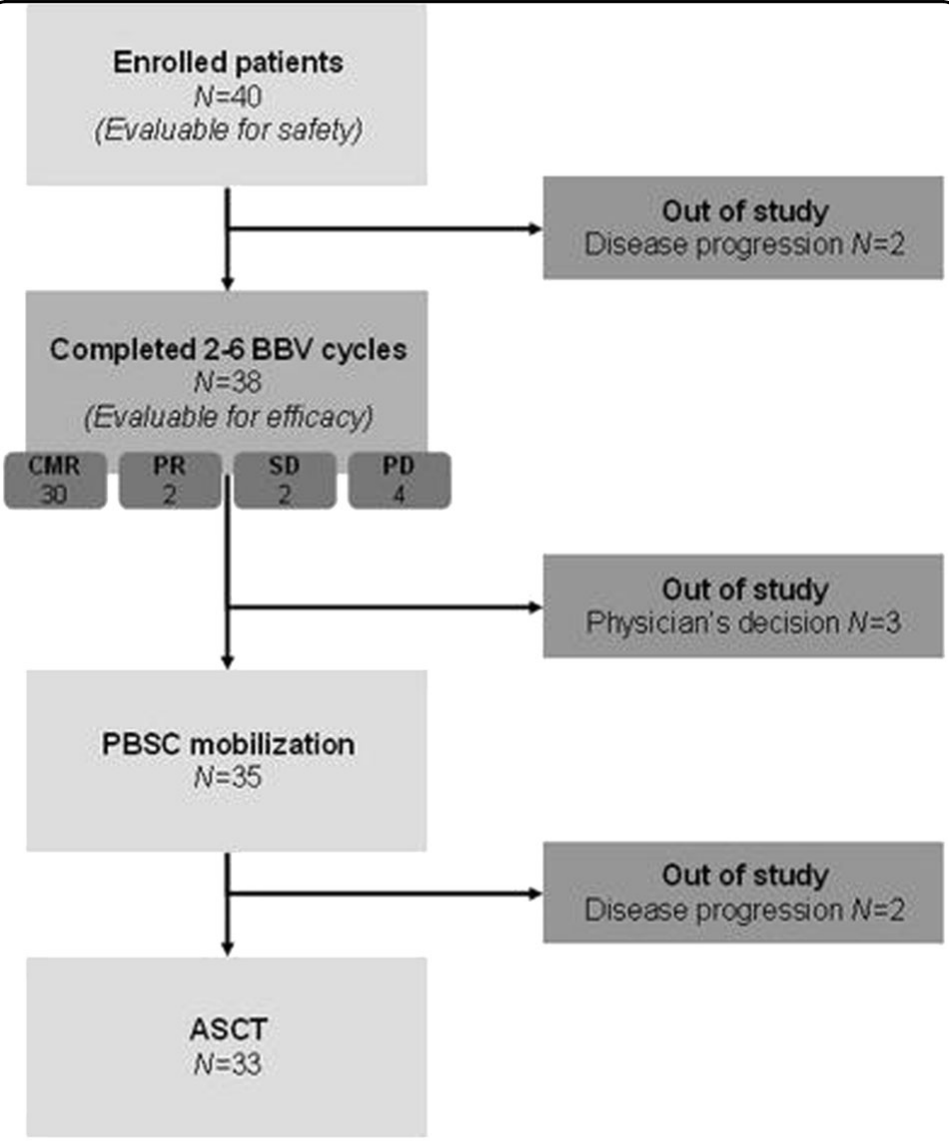
Patient disposition. CMR, complete metabolic response; PR, partial response; SD, stable disease; PD, disease progression; PBSC, peripheral blood stem cells; ASCT, autologous stem cell transplantation.

Thirty-five (92.1%) patients underwent PBSC mobilization. Plerixafor was applied, together with G-CSF, in 12 patients (34.3%). Mobilization and harvest were successful in all cases, with a median CD34^+^ cells/kg yield of 3.2 × 10^6^. Thirty-three patients (86.8%) proceeded to ASCT, all of them obtaining a complete response (CR) after this procedure. ASCT was performed after a median of 4 (range: 2–6) cycles. The two patients who did not proceed to ASCT, although being able to mobilize PBSC, obtained a PR and a disease stability after BBV, but showed a disease progression immediately before the conditioning regimen was started. Patient disposition is summarized in Fig. 2.

### Survival analysis

**Fig. 3:**
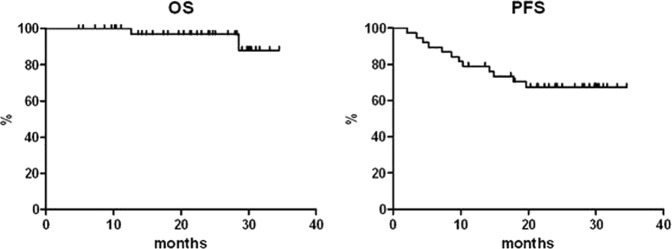
OS and PFS curves plotted for all the patients enrolled in the study.

**Fig. 4:**
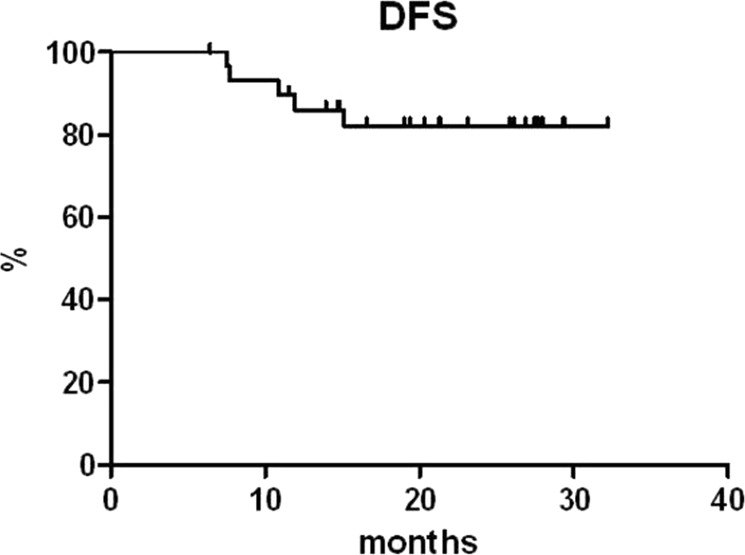
DFS curve for all the patients who obtained a complete metabolic response.

With a median follow-up of 23 months from enrolment into the trial, the OS rates at 1 and 3 years are 100% and 88.1%, respectively. Two patients died during the posttreatment follow-up at 12.6 and 28.6 months, respectively, in both cases as a result of disease progression. The PFS rates are 78.9% and 67.3%, and the DFS rates are 86.1% and 81.9% at 1 and 3 years, respectively (Figs. 3 and 4). Five patients in CMR relapsed during follow-up at 7.5, 7.7, 10.9, 11.9, and 15.1 months since the obtainment of the CR.

### Toxicity

Table 2 summarizes the incidence and the severity of any reported AE. Overall, 41 hematological AEs of any grade occurred during the treatment with BBV, involving 14 patients (35.0%). We observed 13 episodes of neutropenia, 12 of leucopenia, 8 of anemia, and 7 of thrombocytopenia. Febrile neutropenia was reported in one patient: this led to hospitalization, but it rapidly resolved concomitantly with granulocyte recovery. Thirty-eight episodes (92.7%) were judged related to the administered regimen. Only 3 grade IV AEs occurred: they all consisted of transient neutropenia, which resolved within a week and without further complications.

**Table 2:** AEs of any grade.

Toxicity	Patients involved, *N*	Events, *N*	Overall frequency (%)	Grade 1, *N*	Grade 2, *N*	Grade 3, *N*	Grade 4, *N*
*Hematological toxicity*	*14*	*41*	*100*	*16*	*7*	*15*	*3*
Neutropenia	8	13	31.7		2	8	3
Leukopenia	6	12	29.3	2	4	6	
Anemia	8	8	19.5	7	1		
Thrombocytopenia	5	7	17.1	7			
Febrile neutropenia	1	1	2.4			1	
*Extrahematological toxicity*	*37*	*166*	*100*	*87*	*69*	*10*	*0*
Skin reaction	26	46	27.7	19	21	6	
Fever	17	26	15.7	18	7	1	
Nausea	14	24	14.5	16	8		
CMV infection/reactivation	4	9	5.4	1	8		
Diarrhea	4	8	4.8	4	4		
Infusion-related reaction	5	6	3.6	1	5		
Vomiting	4	6	3.6	4	2		
ALT increase	3	5	3.0	4	1		
Flu-like syndrome	4	4	2.4	1	3		
Muscle/joint pain	4	4	2.4	4			
Paresthesia	3	3	1.8	3			
AST increase	2	2	1.2	2			
Dyspnea	2	2	1.2	1	1		
Fatigue	2	2	1.2	2			
Gamma-GT increase	2	2	1.2		1	1	
Pruritus	2	2	1.2	1	1		
Abdominal pain	1	1	0.6	1			
Alkaline phosphatase increase	1	1	0.6		1		
Bronchial infection	1	1	0.6		1		
Candidosis	1	1	0.6		1		
Catheter infection	1	1	0.6			1	
Cold	1	1	0.6		1		
Constipation	1	1	0.6	1			
Heartburn	1	1	0.6	1			
Palpitations	1	1	0.6		1		
Pharyngitis	1	1	0.6		1		
Phlebitis	1	1	0.6	1			
Respiratory failure	1	1	0.6			1	
Sinus tachycardia	1	1	0.6	1			
Vaginal spotting	1	1	0.6		1		
Viral gastroenteritis	1	1	0.6	1			

*CMV* cytomegalovirus, *ALT* alanine transaminase, *AST* aspartic transaminase, gamma-*GT* gamma glutamyl-transpeptidase

In terms of extrahematological toxicity, 166 AEs of any grade occurred during the course of BBV, overall involving 37 patients (92.5%). Among these AEs, 135 (81.3%) were considered therapy related. Most of the AEs were mild (grade I and II) and grade III AEs were observed only in ten cases. No grade IV extrahematological toxicities have been reported. The most frequent AE was represented by skin reactions (reported with the synonyms: skin reaction, erythema, popular eruption, rash, maculopapular rash, and hives), which consisted of 46 episodes, involving 26 patients overall. This AE was judged certainly correlated with the study regimen in seven cases (15.2%), possibly or probably related in 33 cases (71.7%), probably unrelated in three instances (6.5%), whereas it was attributed to other medications in three cases (6.5%). The median duration of skin-related toxicity was 6 (range: 1–96) days; in 19 cases, the duration was shorter than or equal to 3 days, thus indicating a close relationship with the timing of administration of the study drug combination. Seventeen patients experienced fever (26 episodes overall), whose median duration was 3 (range: 1–21) days. In 19 cases, the duration was shorter than or equal to 3 days, which encompassed the 3-days duration of administration of the BBV regimen. Infusion-related reactions (IRR) occurred in five patients (six episodes): notably, they manifested with respiratory failure and dyspnea in one case each. The incidence of BV-induced peripheral neuropathy was indeed rather low: it manifested with grade 1 paresthesias in three patients (1.8%), and it was reversible in all cases.

Seven patients (17.5%) permanently discontinued BBV due to an AE, in six cases because of grade III skin reaction and in one case as a consequence of grade III neutropenia.

Long-term toxic effects are at present unknown: we report no secondary malignancies so far.

## Discussion

Ideally, a first salvage regimen should allow: (i) an effective disease control, which needs to translate into the chance of reinduce high CR rates; (ii) a proper mobilization of PBSC without the use of further chemotherapy in patients for whom ASCT is an option; (iii) a long duration of response in those who are not candidate for a high-dose consolidation, after having obtained an adequate response; and (iv) a good toxicity profile, most of all without myelotoxic events and hopefully avoiding prolonged peripheral cytopenias.

The effective combination of bendamustine and BV in the first salvage setting is supported by a relevant rate of objective responses observed in patients with relapsed or refractory cHL, along with a significant proportion of CMR (84.2% and 78.9%, respectively), stringently confirmed by the application of the Deauville score (Deauville 1–3). The BBV regimen is also a safe alternative to conventional chemotherapy, as it displays limited myelotoxicity and does not impair a subsequent mobilization of PBSC: this is confirmed by the fact that 92.1% of patients could successfully accomplish harvest with a rate of success of 100%. Besides that, however, we cannot exclude a potential toxicity of this regimen on the stem cell itself, as demonstrated by the fact that at least a third of patients required plerixafor to obtain an effective stem cell mobilization in peripheral blood.

Notably, the BBV combination has activity in primary refractory patients, suggesting its potential role in a disease that lacks a proper response to conventional cytostatic drugs. IRR represent the most relevant extrahematological toxicity: albeit observed frequently, they were generally mild and transient, due to a mandatory premedication with steroids and antihistamines, and they were the cause of treatment interruption just in a few cases.

Similar results have been obtained in an earlier report by LaCasce et al. for the same treatment context and in a population with overlapping clinical characteristics^15,18^: a complete response (CR) was observed in 73.6% of patients, with an ORR of 92.5% (according to the 2007 Revised Response Criteria for Malignant Lymphoma^19^), including 85.7% among those with primary refractory cHL. PBSC collection was not impaired either, as most of patients could harvest an adequate amount of CD34^+^ cells at first attempt following G-CSF alone or cyclophosphamide priming and plerixafor, obtaining a median yield of 4.2 × 10^6^/kg. About 73% of the patients enrolled in this trial received a subsequent ASCT after a median of BBV cycles, in comparison to 88% of the patients enrolled in our study, who did not proceed to ASCT only because of disease progression. Similarly to our trial, IRR occurred in 56.4% of patients as a consequence of the combination therapy, therefore requiring a mandatory premedication to be introduced while the study was ongoing: this decreased the severity of IRR, although not clearly reducing their incidence. Importantly, an optional post-ASCT maintenance phase with single-agent BV was planned in the trial by LaCasce et al., which was not part of the treatment in our study: this may have impacted on the survival outcomes of patients not receiving an ASCT consolidation^19^. High remission rates and promising long-term survival results have been equally shown with the same combination in patients failing more than one previous line of therapy, including some heavily pretreated patients^20,21^.

BV has also been combined with conventional chemotherapy schedules, such as ICE (ifosfamide, cytarabine, and etoposide), DHAP (dexamethasone, cytarabine, and cisplatin), and ESHAP (etoposide, prednisolone, cytarabine, and cisplatin): according to preliminary results, all these combinations proved effective in the first salvage setting, with CR rates ranging from 69% to 100%, again without hampering PBSC mobilization and harvesting^22–24^. Very recently, the first chemo-free combination, consisting of BV and the antiprogrammed death 1 (PD1) blocker nivolumab, has been reported in patients with cHL failing induction: the ORR was 82% and the CMR rate 61%, higher than those observed with either agent given alone, and quite similar to what was obtained with BV and bendamustine or ifosfamide/cisplatin-containing regimens^25^. This experience is notable, as it further strengthens the possible role of BV in first salvage settings and provides a sound rationale for chemo-free approaches in relapsed and refractory cHL patients.

Our results, along with previously reported ones, confirm that BV combinations can be profitably exploited in a first salvage setting, where they permit a timely and effective application of the ASCT phase, when indicated^15,18,22–24,26^. Most of all, in respect of the obtainment of a CMR, which is indeed one of the most predictors of post-ASCT survival^6,9^. These results favorably compare to what has been obtained with the bendamustine, gemcitabine, and vinorelbine (BeGeV) regimen, which yielded an ORR of 83% and a CR rate of 73%, with an incidence of grade 3–4 neutropenia, and thrombocytopenia of 14% each and febrile neutropenia of 12%^27^. These results score higher than what was described for ifosfamide/cisplatin-containing regimens and should represent a point of reference for any newer regimen that contemplates agents like BV or anti-PD1 blockers.

BBV and BV-containing first salvage regimes are proposed as a valid alternative to conventional chemotherapy. These regimes certainly represent an attempt to incorporate targeted agents—concomitantly reducing the chemotherapy load—in the treatment of high-risk patients with cHL, and their role appears relevant in those patients who inadequately respond to standard induction. The lack of significant myelotoxicity and the reduced morbidity correlated with prolonged or profound cytopenias could make a regimen like BBV exploitable also in patients, who require an effective salvage although not being candidate to ASCT: this may be inferred by any of the reported experiences, despite none of the studies was specifically addressed to an ASCT-ineligible population.

In conclusion, data suggest that the synergism of bendamustine and BV exhibits promising results in a challenging subpopulation of cHL patients. In the era of new drugs for the treatment of cHL, whether BV-containing regimens can replace conventional chemotherapy deserves further investigation.
